# Determining significance of pairwise co-occurrences of events in bursty sequences

**DOI:** 10.1186/1471-2105-9-336

**Published:** 2008-08-08

**Authors:** Niina Haiminen, Heikki Mannila, Evimaria Terzi

**Affiliations:** 1 HIIT, Department of Computer Science, P.O. Box 68, FI-00014 University of Helsinki, Finland; 2 HIIT, Laboratory of Computer and Information Science, Helsinki University of Technology, FI-02015 TKK, Finland; 3 IBM Almaden Research Center, 650 Harry Road, San Jose, CA 95120, USA; 4 This work was mostly done while the author was at HIIT, University of Helsinki, Finland

## Abstract

**Background:**

Event sequences where different types of events often occur close together arise, e.g., when studying potential transcription factor binding sites (TFBS, events) of certain transcription factors (TF, types) in a DNA sequence. These events tend to occur in bursts: in some genomic regions there are more genes and therefore potentially more binding sites, while in some, possibly very long regions, hardly any events occur. Also some types of events may occur in the sequence more often than others.

Tendencies of co-occurrence of binding sites of two or more TFs are interesting, as they may imply a co-operative role between the TFs in regulatory processes. Determining a numerical value to summarize the tendency for co-occurrence between two TFs can be done in a number of ways. However, testing for the significance of such values should be done with respect to a relevant null model that takes into account the global sequence structure.

**Results:**

We extend the existing techniques that have been considered for determining the significance of co-occurrence patterns between a pair of event types under different null models. These models range from very simple ones to more complex models that take the burstiness of sequences into account. We evaluate the models and techniques on synthetic event sequences, and on real data consisting of potential transcription factor binding sites.

**Conclusion:**

We show that simple null models are poorly suited for bursty data, and they yield many false positives. More sophisticated models give better results in our experiments. We also demonstrate the effect of the window size, i.e., maximum co-occurrence distance, on the significance results.

## Background

Given a set of possible event types, an event sequence is a sequence of pairs (*r, t*), where *r *is an event type and *t *is the occurrence location, or time, of the event. Our focus is on measuring whether the co-localization of occurrences of events of two types is significant in a given sequence.

As an example, consider transcription factor binding sites (TFBS) in a DNA sequence, see [1]. TFBSs occurring close to each other may belong to the same regulatory module. Such modules usually span an interval of 50 – 200 base pairs [2]. These closely located TFBSs could interact in forming larger protein complexes that regulate gene expression. Thus it is of interest to discover which transcription factors (event types) tend to co-occur, and potentially interact, in genomic sequences. Regulatory modules can sometimes be observed from DNA sequences by studying the co-localization of potential TFBS in short sequence windows. This can be done either on a genome-wide scale or in the context of regulatory regions, see for example [3-7]. TFBS co-occurrences have also been used in predicting regulatory regions, see [8]. Given a pair of event types (*r, r'*), there are several possible ways of quantifying their degree of co-occurrence. One can, for example, compute the mean distance from each occurrence of an event of type *r *to the next event of type *r'*, or look at the distribution of such distances. In this paper we measure the co-occurrence of event types *r *and *r' *either by (i) dividing the sequence into non-overlapping windows of a fixed length *w *and counting the number of windows that contain at least one event of type *r *and at least one event of type *r'*, or by (ii) counting the number of events of type *r *that are followed by at least one event of type *r' *within distance *w*, or by (iii) counting the number of events of type *r *that are followed or preceded by at least one event of type *r' *within distance *w*. These co-occurrence scores are used because of their simplicity and intuitiveness; other co-occurrence scores could be used as well. The point we make here is that the numerical value of such a score in itself is not very informative.

In order to determine the significance of a co-occurrence score, we need a null model to estimate the distribution of the score values and then decide the significance of an individual value. We define three such null models. These models apply to any co-occurrence score, not just the ones used here. Null co-occurrence score distributions for TFBSs have been estimated by Levy, Hannenhalli & Workman [3], Hannenhalli & Levy [4], and Klein & Vingron [8] by performing randomization experiments. We introduce an additional null model and compare it to those that have been suggested before. Our experimental results on synthetic event sequences demonstrate that our novel null model provides more accurate results than previously suggested models in certain scenarios.

**Figure 1:**
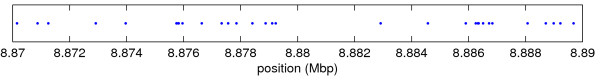
**Example of burstiness in a DNA sequence**. A 20 kbp region from the chromosome 1 sequence described in Results and Discussion, showing locations of matches to the Jaspar motifs. Short bursts are visible, e.g., 6 closely located matches around 8.876 Mbp.

Good null models take into account the global event sequence structure, including the tendency of events to occur in bursts (also called clumps, or clusters). For example, in gene-rich DNA regions potential binding sites may occur very densely, while in gene-poor areas the event density can be considerably smaller. An example of a bursty DNA event sequence is shown in Figure 1. The figure shows potential binding sites in a 10 kbp region in chromosome 21, featuring a burst of length 290 bp where potential TF binding sites occur more densely than elsewhere in the sequence. The DNA sequence and the method for obtaining the binding sites are described in more detail in Results and Discussion. We show that a simple model that is equivalent to the standard *χ*^2 ^significance test is poorly suited for such bursty data, yielding many false positives. More sophisticated models, on the other hand, make it possible to find exactly the planted co-occurrences in synthetic data as significant, and not many more.

The main contribution of this work is in the formal definition of null models for event sequences and demonstrating the need for different null models. We discuss and compare the performance of the null models on synthetic event sequences and on data consisting of potential TFBS occurrences in a human DNA sequence. We study the effects of the distance *w *and the *p*-value threshold on the number of TF pairs that are found significant.

## Methods

### Sequences of events

Consider a data sequence (e.g., a DNA sequence or time series) containing *n *possible locations {1,..., *n*} where events can occur. Assume that there is a set *R *of event types, and that *m *events occur in the sequence. An event sequence *S *= {*s*_1_,..., *s*_*m*_} consists of pairs *s*_*i *_= (*r*_*i*_, *t*_*i*_), where *r*_*i *_∈ *R *and *t*_*i *_∈ {1,..., *n*}. We use *T *= {*t*_1_,..., *t*_*m*_} to denote the set of locations where events occur in *S*. For example, the data sequence can be a DNA sequence of length *n *in which *m *potential transcription factor binding sites occur. The types would then correspond to specific transcription factors, and the locations to positions at which the binding sites appear in the sequence.

### Co-occurrence scores

**Figure 2:**
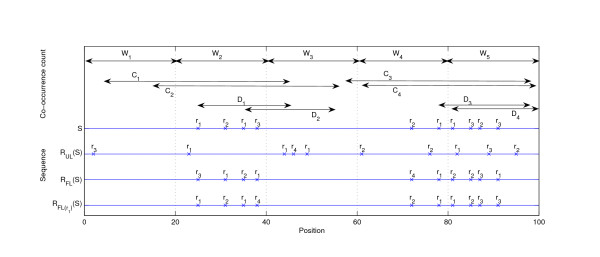
**Illustration of the null models**. An event sequence *S *of length *n *= 100 with event types *r*_1_, *r*_2_, *r*_3_, *r*_4 _and illustration of null models UL, FL, and FL(*r*) w.r.t. *S *with distance parameter *w *= 20. The sequence regions from which the *W*, *C*, and *D *scores are computed w.r.t. sequence *S *and event type *r*_1 _are shown in the top half of the figure (*n/w *= 5 regions for the *W *score, and *c*(*r*_1_) = 4 regions for the *C *and *D *scores). Models FL and FL(*r*) keep the locations of the events fixed, while UL randomly assigns new locations. In addition, here FL(*r*_1_) keeps the labels of events of type *r*_1 _fixed. All methods maintain the total number of events of each type. The co-occurrence counts for the pair (*r*_1_, *r*_2_) in the original sequence are *W *(*r*_1_, *r*_2_, *S*) = 3, *C*(*r*_1_, *r*_2_, *S*) = 4, and *D*(*r*_1_, *r*_2_, *S*) = 3. For the randomized sequences the counts are *W *(*r*_1_, *r*_2_, *R*_UL_(*S*)) = 1, *C*(*r*_1_, *r*_2_, *R*_UL_(*S*)) = 3, *D*(*r*_1_, *r*_2_, *R*_UL_(*S*)) = 3, *W *(*r*_1_, *r*_2_, *R*_FL_(*S*)) = 2, *C*(*r*_1_, *r*_2_, *R*_FL_(*S*)) = 4, *D*(*r*_1_, *r*_2_, *R*_FL_(*S*)) = 2, *W*(*r*_1_, *r*_2_, $R_{\text{FL}(r_{1})}$ (*S*)) = 3, *C*(*r*_1_, *r*_2_, $R_{\text{FL}(r_{1})}$ (*S*)) = 4, and *D*(*r*_1_, *r*_2_, $R_{\text{FL}(r_{1})}$ (*S*)) = 3.

Given the sequence *S *= {(*r*_1_, *t*_1_), (*r*_2_, *t*_2_),...,(*r*_*m*_, *t*_*m*_)}, let *c*(*r*) be the number of times event type *r *occurs in the sequence *S*, and denote *f*(*r*) = *c*(*r*)*/m*. Divide the underlying *n *possible locations into non-overlapping windows of width *w*. The *window count W*(*r*, *r'*, *S*) for event types *r *and *r' *is the number of windows in which at least one event of type *r *and at least one event of type *r' *occur. Thus the values of *W*(*r, r', S*) are in [0, ⌈*n/w*⌉]. The *co-occurrence count C*(*r, r', S*) is the number of events of type *r *that are followed or preceded by at least one event of type *r' *within distance *w*. The values of *C*(*r, r', S*) are in [0, *c*(*r*)]. The *directed co-occurrence count D*(*r, r', S*) is the number of events of type *r *that are followed by at least one event of type *r' *within distance *w*. The values of *D*(*r, r', S*) are also in [0*, c*(*r*)]. See [9] for similar scores. When the pair of event types (*r, r'*) and the sequence *S *are implied by the context, we use the notation *W*, *C*, and *D*. See Figure 2 for an illustration of the windows and corresponding co-occurrence scores.

Note that the total number of event type pairs is *O*(|*R*|^2^), and recall that *m *is the number of events in the sequence. The *W *score is the computationally most efficient to calculate; it takes time *O*($m+\frac{n}{w}$|*R*|^2^) to obtain the 0/1 occurrence count of each event type in each window, and to multiply these counts for each event type pair in each window. Computing the *C *score takes at most time *O*(*mk*|*R*|), where *k *is the maximum number of events within distance *w *from any event. This is because for each event we need to check the 0/1 occurrence of each type in its neighborhood; the worst case time complexity is therefore *O*(*m*^2^|*R*|). The *D *score has the same worst case time complexity as the *C *score. However, now *k *denotes the maximum number of events following any event within distance *w*, which is expected to be less than the *k *for the *C *score.

### Null models

Here we describe the null models that we use when computing the significance of the *W*, *C*, and *D *scores obtained on the input sequence. There are three models: the *uniform locations *(UL) model, the *fixed locations *(FL) model and the *fixed locations fixed event type *(FL(*r*)) model. Below we explain how to generate randomized versions of a given event sequence *S *according to these models:

#### Uniform locations UL

Generate a sequence *R*_UL_(*r*) by creating *c*(*r*) events (*r, t*_*i*_), *i *∈ 1,..., *c*(*r*), where each *t*_*i *_is selected uniformly at random from {1,..., *n*}. Note that several events might have the same location. The randomized version of *S*, *R*_UL_(*S*), is a union of the *R*_UL_(*r*). A similar model is applied in, e.g., [3].

#### Fixed locations FL

The randomized sequence *R*_FL_(*S*) is the sequence {(*q*_1_, *t*_1_),...,(*q*_*m*_, *t*_*m*_)}, where the event types *q*_*i *_are selected independently at random with probabilities *f*(·). That is, the event locations are kept fixed, and the event types are assigned at random according to their frequencies in the original sequence. A similar model is applied in, e.g., [4,8].

#### Fixed locations fixed event type FL(*r*)

Given a sequence *S *and an event type *r*, the randomized sequence *R*_FL_(*r* (*S*) is defined as *R*_FL_(*S*), with the exception that the occurrences of events of type *r *are kept unchanged. That is, type *r *is assigned for those locations *t*_i _for which *r*_i _= *r*, and the types for all other event locations are assigned from *R*\*r *according to their frequencies *f*(·). We are unaware of any previous studies on this type of a null model.

An example of the randomized sequences *R*_UL_(*r*), *R*_FL_(*S*) and *R*_FL_(*r* (*S*) is given in Figure 2.

### Empirical *p*-values

For a given sequence *S *and a null model *M *∈ {UL, FL, FL(r)} we compute the empirical *p*-value of the *W *(or *C*, *D*) score for event types *r *and *r' *as

*p*_*W *_(*r*, *r'*, *M*, *S*) = Prob[*W *(*r*, *r'*, *S*) ≤ *W *(*r*, *r'*, *R*_*M*_(*S*))].

In other words, we compute the fraction of randomizations in which the *W *(or *C*, *D*) score for the randomized sequence *R*_*M*_(*S*) exceeds the *W *(or *C*, *D*) score for the original sequence *S*. When simultaneously testing multiple hypotheses, methods for controlling the false discovery rate can be applied [10].

## Results and Discussion

Here we describe our experiments on synthetic and real event sequences. We also discuss the implications of our results on potential transcription factor binding site occurrences.

### Synthetic data

Our experimental study aims to evaluate the different null models with respect to two diagnostics: (1) whether they find the planted co-occurrence patterns and (2) whether they are able to discard non-existing co-occurrence patterns (false positives). To study the null models w.r.t. these two diagnostics, we generated uncorrelated, correlated, and directed sequences as described below. We varied the burstiness in the generated sequences, and in some of them we planted a pattern of frequent co-occurrence between two event types.

The generative model for our data is as follows. We randomly divide the sequence into some number of *sparse *and *dense *segments. In each position in the sparse segments, an event of any type occurs with probability *p*_1_. For the dense segments, the corresponding probability is *p*_2 _> *p*_1_; more events are expected to occur in the dense segments. The lengths of the dense segments are chosen uniformly at random from [100, 200], and 50 such segments are randomly positioned in the sequence (making sure they do not overlap).

We generated five types of sequences using this model. The sequences of the first type, *uncorrelated*, do not contain any correlations between event types; after deciding the positions for the events, we choose the type of each event uniformly at random. The sequences of the second type, *correlated*, contain an undirected frequent pattern of co-occurrence between two types of events, *a *and *b*, denoted (*a, b*). That is, the type of each event is again chosen uniformly at random, except that every time an event of type *a *occurs, it is with high probability followed by an event of type *b*, and the same holds for *b *followed by *a*. The third type of data, *directed*, contains a directed frequent co-occurrence pattern denoted *a *→ *b*. In this case the pattern *a *followed by *b *is planted in the sequence (and not *b *followed by *a*).

For the *distinct correlated *and *distinct directed *sequences we generated sparse and dense segments as before, but in some number of the dense segments (chosen uniformly at random from [5, 25]) a co-occurrence pattern between *a *and *b *was planted. In the distinct directed sequences this means that in some number of the dense segments whenever *a *occurs, it is with high probability followed by *b*. In the corresponding dense segments in the distinct correlated sequences, *b *is also followed by *a *with high probability. Thus the co-occurrence patterns were only planted in some distinct dense segments. In the remaining dense segments and in the sparse segments all types occur with equal probability.

In Table 1 we show the results for the experimental diagnostics (1) and (2). For the experiments we used a fixed set of parameter values: 10 event types, sequence length 10^5^, *p*_1 _= 0.01 and *p*_2 _= 0.1. We report the number of pairs of types whose co-occurrence score is significant (*p *≤ 0.01) when *w *= 50. The *p*-values for all null models were obtained by performing the corresponding randomizations and computing empirical *p*-values based on the results. In the case of the diagnostic (1) the co-occurrence of *a *and *b *is significant in sequence *S *according to score *L *= {*W, C, D*} if either *L*(*a, b, S*) or *L*(*b, a, S*) is found significant. That is, e.g., the co-occurrence score for *a *and *b *is significant either in randomizations where the locations of events of type *a *are fixed, or in randomizations when the locations of events of type *b *are fixed.

**Table 1:** Number of significant pairs in synthetic data

Dataset	Number of significant pairs									
	*W*			*C*			*D*			
				
	UL	FL	FL(*r*)	UL	FL	FL(*r*)	UL	FL	FL(*r*)	

1. Uncorrelated	39	0	0	54	1	0	54	1	0	
2. Correlated	33	1	2	54	2	2	53	2	2	
3. Directed	38	1	1	54	1	1	54	2	1.5	(1)
4. Distinct correlated	38	1	1	54	1	1	54	1	1	
5. Distinct directed	39	1	1	54	1	1	54	1	1	
										
	Number of randomizations where (*a, b*) found significant									

1. Uncorrelated	85	1	0	97	0	0	90:92	1:1	0:1	
2. Correlated	100	100	100	100	100	100	100:100	100:100	100:100	
3. Directed	100	93	99	100	88	99	100:98	100:1	100:2	(2)
4. Distinct correlated	94	34	35	97	33	34	95:94	35:33	36:33	
5. Distinct directed	93	29	31	99	5	17	96:97	31:5	33:0	

(1) Median number of pairs of event types, over 100 randomly generated sequences, whose co-occurrence score is significant. Results are shown for five types of synthetic datasets. (2) The number of randomizations in which the planted pair (*a, b*) is found significant. UL, FL, and FL(*r*) correspond to the null models, and *W*, *C*, and *D *to the window, undirected, and directed co-occurrence scores. For the *D *score, the two values *s*_1_: *s*_2 _denote the number of randomizations in which (*a *→ *b*) and (*b *→ *a*) are found significant. The empirical *p*-values are based on 1000 randomizations. Results are shown for *p*-value threshold 0.01, with 10 event types, *w *= 50, burst lengths in [100, 200], sequence length 100000, and parameter values *p*_1 _= 0.01, *p*_2 _= 0.1, with 50 bursts per sequence. For the datasets 4 and 5, the number of bursts containing correlations was randomly chosen from [5, 25].

From Table 1 we can see that the FL and FL(*r*) null models yield very good results on the first three datasets: in nearly all randomizations the planted co-occurrence and not many more co-occurrences are found significant. FL(*r*) finds the planted co-occurrence slightly more often than FL for dataset Directed with the *C *score, and it finds slightly fewer false positives with the *D *score.

Note that as there are 10 event types, there are (10^2 ^- 10)/2 + 10 = 55 pairs of types (including pairs of type (*a*, *a*)), and one of them is truly significant. Thus with *p*-value threshold 0.01 we expect to see about 0.54 false positives. Using the UL null model we find the true co-occurrence pattern. However, this model also finds a very large number of false positives, close to the total number of pairs.

For the last two datasets in Table 1, the FL(*r*) model yields the best results: it finds the planted co-occurrence pattern more successfully than FL, while yielding a smaller number of false positives when compared to UL or FL. We conclude that the FL(*r*) null model yields results close to FL on uncorrelated, correlated and directed sequences, and it is the best model to use when the co-occurrence pattern between a pair of event types occurs in a distinct subset of the bursty regions in the sequence.

The probabilities *p*_1 _and *p*_2_, as well as the lengths of the bursts have an effect on the burstiness of the data. In addition to these parameters, the length *w*, the number of event types, and the *p*-value threshold also affect the results for each null model. In more extensive tests, we varied the lengths of the bursts; we generated 100 sequences whose burst lengths were bl_1_, randomly chosen from [50, 100], and 100 sequences with burst lengths bl_2_, randomly chosen from [100, 200]. We computed the empirical *p*-values using *w *= {50, 100, 200, 500} with 1000 randomizations for these 200 sequences. The number of event types was again 10 while the sequence length was 100 kbp. We made the following observations about the datasets of types uncorrelated, correlated, and directed for *p*-value thresholds 0.01 and 0.001 (data not shown): the UL model gives the largest number of false positives in each parameter setting, and the FL and FL(*r*) models find the planted frequent co-occurrence pattern for at least 90% of the generated sequences for almost all parameter settings. In the cases where the FL and FL(*r*) found the planted pattern for less than 90% of the sequences (this occurred when *w *= 500 both for bl_1 _and bl_2_, with both *p*-value thresholds), the UL model found it significant an even fewer number of times. We thus conclude that the FL and FL(*r*) models are successful in finding frequent co-occurrence patterns for a variety of burst lengths and values of *w*.

### TFBS motif occurrences

Potential binding sites of transcription factors are an example of biological event sequences where co-occurrence patterns and burstiness occur. We applied our techniques on 10 Mbp regions from human chromosomes 1–10 [11] (NCBI 36 assembly), where we identified potential binding sites as matches to known transcription factor binding motifs. The regions 30 – 40 Mbp were used for chromosomes 1–9, and 20 – 30 Mbp for chromosome 10, to avoid the centromere region. This dataset contains genome regions with different characteristics (e.g., C+G and gene densities), while being compact enough to be efficiently studied with several null models and window sizes. The motifs we consider are from the Jaspar collection [12] (Jaspar Core), all 138 motifs in the 2008 build. In these sequences we identified all matches for each Jaspar transcription factor (TF) matrix by the PoSSuMsearch program [13]. The threshold for a match was set with *p *≤ 10^-5^, yielding approximately 30000 matches for each 10 Mbp sequence. With this *p*-value threshold, some Jaspar motifs are not specific enough to yield any matches, resulting in 115 possible motifs, or, event types in the event sequence.

Thus the event sequence consists of pairs of the format (*position, type*), where *position *marks the start of the match, and *type *the index of the TF. The starting position of a match is defined as its smallest distance from the start of the sequence, i.e., a match spanning sequence positions 100 to 110 has starting position 100. The match for a given TF on either strand is counted as an event of the same type; thus strand-specificity is not considered. The number of matches per TF per sequence in the sequences ranges from 3 (MIZF) to 4029 (HMG-IY). We noticed that the number of matches in each 100 kbp region tends to be larger in G+C and gene rich regions, the number of matches per 100 kbp ranging from 200 to 400 (results not shown). Therefore the assumption of regions with varying event density holds for this data. In the following we study the sequence for distances *w *ranging from 100 bp to 500 bp.

Since some of the Jaspar motifs are structurally similar, overlapping matches for two factors can occur in the event sequence. To prune out this source of false co-occurrence patterns, we processed the event sequence for each factor separately. When studying the significance of a factor pair (*a*, ·), we disregarded all events closer than distance *d *to the location of a given occurrence of type *a*. Experimentally we found that *d *= {10, 20, 50} produced almost identical results, while *d *= 0 gave many false co-occurrence patterns due to overlapping matches. The longest motif in our collection was of length 20, which we chose to use as the value of *d *in the experiments. We also pruned out exactly overlapping matches for the same TF (matches occurring on forward and reverse complement strands simultaneously). The *W *score was not used in these experiments, as the implementation that disregards closely occurring matches would not be any more efficient than those for the *C *and *D *scores.

The number of pairs in each sequence with significant co-occurrence scores are shown in Table 2 The table shows results for the distance *w *= 300 bp and *p*-value thresholds *p *∈ {0.01, 0.001}. All empirical *p*-values are over 1000 randomizations. The co-occurrence of a pair (*a*, *b*) is significant in sequence *S *if either *C*(*a*, *b*, *S*) or *C*(*b*, *a*, *S*) is found significant. The numbers for different chromosomes are quite similar, indicating that the chosen regions are similar in their sequence composition and tendency to contain matches to pairs of Jaspar motifs. The results show that the UL model yields more significant pairs than the other models, in one case over four times as many (chromosome 2, *D *score). Lowering the *p*-value threshold reduces the number of pairs somewhat, as is expected, but the choice of a *p*-value between 0.01 and 0.001 does not radically alter the magnitude of the TF pairs that are found significant. The effect of the window size *w *is shown, as an example, for chromosome 1 in Table 3. Increasing the window size from 300 bp to 500 bp only slightly increases the number of significant pairs, and the number is even reduced in some cases (FL(*r*) model, *p *≤ 0.001). This indicates that many choices of *w *yield a consistent number of pairs whose co-occurrence is significant on that distance scale.

**Table 2:** Number of significant pairs in chromosome data

	*C*						*D*					
		
	UL		FL		FL(*r*)		UL		FL		FL(*r*)	
						
chr	0.01	0.001	0.01	0.001	0.01	0.001	0.01	0.001	0.01	0.001	0.01	0.001
1	134	68	108	60	96	41	143	71	123	62	87	40
2	138	73	36	29	84	41	153	83	33	22	87	35
3	146	90	118	62	90	47	162	87	131	71	98	45
4	192	120	116	60	104	53	217	122	110	64	107	50
5	138	85	90	60	98	51	146	79	92	51	88	37
6	146	83	119	60	107	59	165	79	131	58	112	40
7	147	78	86	52	87	37	161	93	117	62	100	43
8	130	76	96	57	79	30	159	86	115	65	93	39
9	200	119	158	101	125	58	243	125	196	102	137	54
10	154	100	126	70	93	45	164	97	137	75	103	50

Number of significant pairs for 10 Mbp regions in human chromosomes 1–10. Results are shown for window sizes *w *= 300 and two *p*-value thresholds, for null models UL, FL, and FL(*r*), for co-occurrence scores *C *and *D*. Minimum distance parameter *d *= 20 bp. Number of event types is 115, and thus the total number of undirected pairs is (115^2 ^- 115)/2 + 115 = 6670 and number of directed pairs is 115^2 ^= 13225.

**Table 3:** Number of significant pairs per window size

	*C*						*D*					
		
	UL		FL		FL(*r*)		UL		FL		FL(*r*)	
						
*w*	0.01	0.001	0.01	0.001	0.01	0.001	0.01	0.001	0.01	0.001	0.01	0.001
100	90	44	67	31	70	23	102	55	89	40	63	20
300	134	68	108	60	96	41	143	71	123	62	87	40
500	151	80	130	67	107	39	171	83	142	72	106	37

Number of significant pairs for 10 Mbp regions in human chromosome 1. Results are shown for window sizes *w *∈ {100, 300, 500} and two *p*-value thresholds, for null models UL, FL, and FL(*r*), for co-occurrence scores *C *and *D*. Minimum distance parameter was *d *= 20 bp. Number of event types is 115, and thus the total number of undirected pairs is 6670 and number of directed pairs is 13225.

Table 4 shows the number of FL- and FL(*r*)-specific pairs for each chromosome, and the number of pairs that are found significant for both null models (*w *= 300, *p *≤ 0.001). The results show that there are quite many pairs that both models find significant. Overall, FL reports more pairs than FL(*r*). It is clear that each model reports a different list of significant pairs, though many of the pairs are shared between the models. Thus it makes a difference which null model one uses in deciding the significance of the co-occurrence of a pair of event types. The complete lists of significant pairs for both models with these parameters are given in Additional file 1.

**Table 4:** Differences between FL and FL(*r*) significant pairs in chromosome data

chr	FL	both	FL(*r*)
1	28	38	3
2	11	21	20
3	33	34	13
4	30	34	19
5	26	38	13
6	25	42	17
7	25	30	7
8	35	27	3
9	57	49	9
10	35	39	6

The number of significant pairs (*p *≤ 0.001) in chromosome data according to *C *score and null models FL and FL(*r*). Parameters used are *w *= 300 bp, minimum distance *d *= 20 bp. The number of FL- and FL(*r*)-specific pairs is shown, and the number of pairs that both models find significant.

An example of the pairs that are found significant is shown in Table 5. The significant TF pairs with the 20 highest *C *scores among all the studied chromosome sequences are listed in the table for *w *= 300, according to the *FL*(*r*) randomization. The full names of the TFs are given in Additional file 2. As Table 3 shows, the *C *score for a pair is not directly related to the number of times each TF occurs in the sequence, e.g., pair (RREB1, SP1) has a higher score than (HMG-IY, STAT1) whose TFBSs occur more frequently. In total there are 241 unique significant pairs (with *p*-values *p *≤ 0.001) with these parameters among all the chromosome sequences. Typically the pairs are also found significant by the FL model. Some pairs are found significant in only one chromosome sequence, e.g., (HMG-IY, ESR1) in chromosome 4, and some pairs in all sequences, e.g., (FOXI1, HMG-IY). This would indicate that there are TF pairs whose potential binding sites have a significant tendency for co-occurrence across the genome, while some pairs may only show that tendency in specific genome regions.

**Table 5:** Significant pairs in chromosome data

chr	TF 1	TF 2	# TF 1	# TF 2	*C*	num	FL	ref
1	MA0042, FOXI1	MA0045, HMG-IY	1656	3958	550	10	Y	
1	MA0041, Foxd3	MA0045, HMG-IY	1643	3958	547	10	Y	
9	MA0045, HMG-IY	MA0119, TLX1-NFIC	3771	985	458	7	Y	
2	MA0073, RREB1	MA0079, SP1	1968	447	317	6	Y	[16]
1	MA0045, HMG-IY	MA0088, Staf	3958	583	180	4	Y	
9	MA0045, HMG-IY	MA0137, STAT1	3771	642	173	3	Y	
4	MA0045, HMG-IY	MA0079, SP1	2661	744	170	2	Y	[17]
9	MA0042, FOXI1	MA0119, TLX1-NFIC	1439	985	170	4	Y	
6	MA0045, HMG-IY	MA0082, SQUA	4029	579	164	2	N	
9	MA0003, TFAP2A	MA0073, RREB1	856	1756	131	4	Y	[18]
1	MA0029, Evi1	MA0045, HMG-IY	856	1756	131	4	Y	
4	MA0045, HMG-IY	MA0112, ESR1	465	3958	129	2	Y	[19]
9	MA0041, Foxd3	MA0119, TLX1-NFIC	2661	605	121	1	N	
9	MA0022, dl_1	MA0045, HMG-IY	1162	985	118	4	Y	
1	MA0045, HMG-IY	MA0049, hb	365	3771	110	4	Y	
9	MA0003, TFAP2A	MA0123, ABI4	3958	387	110	1	N	
4	MA0073, RREB1	MA0123, ABI4	856	355	107	10	Y	
4	MA0045, HMG-IY	MA0048, NHLH1	1971	303	103	4	Y	
4	MA0079, SP1	MA0119, TLX1-NFIC	744	1013	102	1	Y	
6	MA0073, RREB1	MA0138, REST	1868	615	100	1	Y	

The significant pairs (*p *≤ 0.001) in chromosome data with the highest *C *scores. The parameters used are *w *= 300 bp, minimum distance *d *= 20 bp, and significance is determined according to the *FL*(*r*) null model for the *C *score. There are in total 241 unique significant pairs in chromosomes 1–10 with these parameters. The chromosome where the *C *score is highest, and the total number of times that each TF occurs in the corresponding chromosome are given, as well as the *C *score. The following two columns state the number of chromosomes (1–10) in which the pair is significant, and if the pair is significant according to the FL null model. The last column gives a reference when one exists. Additional file 2 contains the full names of the factors.

**Figure 3:**
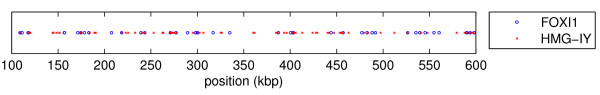
**Co-localization of FOXI1 and HMG-IY**. A visualization of the potential binding sites for TFs FOXI1 and HMG-IY in a 500 kbp subsequence from the chromosome 1 sequence described in Results and Discussion. In several cases the starting positions of the TF matches are located within a very short distance from each other.

As an example, the co-localization of the pair with the highest *C *score, (FOXI1, HMG-IY), is visualized in Figure 3, for a subsequence from chromosome 1. We compared the locations of the matches in this sequence region to Ensembl gene annotations , but did not observe tendencies for the pairs to occur in, e.g., upstream regions. Indeed, a recent study by Blanchette et al. [7] found that their predicted regulatory modules also show enrichment near the 3' end of genes and in regions far from genes. Further studies would be required to make conclusions about the genomic regions where the significant pairs are located.

We used the Chilibot [14] website to search for PubMed abstracts where the pairs of TFs in Table 5 occur. The search results are given as references in the last column of the table. We also searched the TRANSCompel [15] database for interactions between the pairs for which no PubMed results were found, but found no further evidence of interaction. This can in part be due to different naming conventions in the Jaspar and TRANSFAC databases. The Chilibot search tool, on the other hand, is incorporated with a database of synonymous terms. The references shown in Table 5 show interactions or connections that have been observed between the respective transcription factors. For 4 out of the 20 pairs with highest *C *scores, such references were found. The remaining pairs may also interact in a variety of ways, but we found no reported connections between them by searching related literature.

The potential binding sites for certain pairs of TFs, e.g., those pairs listed in Table 5, show a statistically significant tendency to occur in the same short regions in the studied chromosome segment that covers many genes and intergenic regions. This could be due to similarities in the DNA sequence composition near their preferred binding sequences. However, we have eliminated the possibilities that the preference would be due to overlapping motif matches (by pruning out events occurring closer than distance 20 from each other), or the tendency for many matches to occur globally in the same regions, i.e., the burstiness effect (by computing the significance according to the FL and FL(*r*) null models).

## Conclusion

In this paper we formally defined a number of null models, against which the significance of co-occurrence between a pair of event types can be determined in a sequence of events. The models formalize and extend the work of Levy *et al*. [3] and Hannenhalli & Levy [4]. Furthermore, we showed how to empirically estimate the *p*-values of co-occurrence significance with respect to these null models and natural measures of undirected and directed co-occurrence. The null models and co-occurrence scores were shown relevant and practical on real data consisting of potential transcription factor binding sites.

We observed that for bursty data, such as TFBS occurrences, those null models that do not take the burstiness into account falsely determined co-occurrences between many pairs as significant. On the other hand, models that take the event locations into account performed well on both simulated and real data, finding significant tendencies for co-occurrence between some TFBSs. Our method for discovering significant co-occurrences between directed pairs of event types also performed well in practice.

An interesting and important direction for applying these type of significance tests would be the promoter regions of co-regulated genes. The null models and corresponding *p*-value computations can also be applied in other areas where co-occurrences of certain location- or time-dependent features is of interest.

## Authors' contributions

All authors participated in the design of the study and provided significant contributions to the content. All authors were involved in writing and revising the manuscript.

## Supplementary Material

Additional file 1 **Significant pairs**. Significant pairs according to the FL and FL(*r*) null models and *C *score in 10 Mbp regions from chromosome 1–10, with window length *w *= 300, minimum distance *d *= 20, and empirical *p*-value *p *≤ 0.001. Each row contains the following 5 columns: chromosome, TF 1 (e.g., 45 corresponds to Jaspar matrix MA0045), TF 2, *C *score, 0 if the pair (TF 1, TF 2) is significant according to FL, or 1 if it is significant according to FL(*r*). The matrix names are available from the Jaspar database download page: Click here for file

Additional file 2 **Full names of TFs in Table 5**. The full names of transcription factors appearing in Table 5. Click here for file
